# The Prevention of Smallpox

**Published:** 1905-02

**Authors:** 


### THE PREVENTION OF SMALLPOX.

Dr. Benjamin Lee, secretary of the Pennsylvania State Board of Health, points out in his annual report, just submitted, that during the year ended November 1, there were reported in the State 5,172 cases of smallpox, with 521 deaths, which is in marked contrast with the experience of the German army, in which, since vaccination and revaccination were made compulsory, thirty years ago, there has not been a single death from smallpox. In the city of Philadelphia, as a result of an active vaccination crusade, the number of cases of smallpox has been reduced from 396 in December, 1903, to 26 in June, 1904, and in Pittsburg from 537 cases in October, 1903, to 15 in March, 1904. At the present time there are only 42 cases throughout the entire State.—*Medical Record.*

